# The epidemiology of adults' eyelid malignancies in Germany between 2009 and 2015; An analysis of 42,710 patients' data

**DOI:** 10.1177/11206721221125018

**Published:** 2022-11-04

**Authors:** Ahmad S Alfaar, C Nathanael Suckert, Matus Rehak, Christian Girbardt

**Affiliations:** 1 Department of Ophthalmology, Universitätsklinikum Leipzig, Leipzig, Germany; 2 Experimental Ophthalmology, Charité – Universitätsmedizin Berlin, Berlin, Germany; 3 Department of Ophthalmology, University Hospital of Ulm, Ulm, Germany

**Keywords:** Eyelid malignancies, epidemiology, basal cell carcinoma, squamous cell carcinoma, melanoma

## Abstract

**Purpose:**

We report the incidence of malignant tumors of the eyelid in Germany between 2009 and 2015.

**Methods:**

Data pertaining to the period between 2009 and 2015 were extracted from the German Cancer Registry. The International Classification of Diseases for Oncology-3 codes for tumors of the eyelid or canthus were used to identify incidence rates and survival probabilities. Crude and age-standardized incidence rates (ASR) were calculated by age, year, and gender and the individual federated states. Survival was calculated using the Kaplan-Meyer and Life tables methods, and COX-Regression was used to calculate hazard ratios for overall and cancer-specific survival.

**Results:**

This study examined data pertaining to 42,710 patients who had been diagnosed with malignant tumors of the eyelid. Basal cell carcinoma was by far the most common tumor of the eyelid (87.1%), followed by squamous cell carcinoma (10.1%) and malignant melanoma (1.1%). ASRs of basal cell carcinoma, squamous cell carcinoma, and melanoma were 65.1, 7.49, and 0.83 per million, respectively. Sebaceous cell carcinoma did not appear to be of high prevalence in Germany. Cancer-specific survival was generally high, exceeding 95%. The overall survival of patients with melanoma was considerably lower than those with other cancers. Both survival rates were inferior to that of basal cell carcinoma (74.9%). Cancer-Specific survival at five years for all groups exceeded 95%.

**Conclusions:**

The incidence rates of malignant lid tumors match that of the European countries but shows a different pattern than Asian countries.

## Précis

The incidence of Basal cell carcinoma in Germany is 65.1 per million, followed by squamous cell carcinoma with 7.49 and malignant melanoma with 0.83 per million in a distribution different from east and south Asian countries.

## Highlights

- No previous studies have investigated the incidence of malignant tumors of eyelid at the national level in Germany.
- In the period between January 2009 and December 2015, 42,710 patients were diagnosed with malignant tumors of the eyelid in Germany.
- Basal cell carcinoma was the most common eyelid cancer (87.1%), with 65.1 per million, followed by squamous cell carcinoma (10.1%) with 7.49 and malignant melanoma (1.1%) with 0.83 per million.
- Sebaceous cell carcinoma was a rare malignancy among eyelid malignancies.
- Cancer-specific survival was generally high, exceeding 95%; cases with melanoma showing the lowest survival rates.
- Overall survival of squamous cell carcinoma reached 58.3% and malignant melanoma 54.8%, both inferior to basal cell carcinoma (74.9%).

## Introduction

The incidence of skin tumors has been increasing worldwide.^1^ Although the eyelids represent less than 1% of the total body surface, periocular malignancies represent 5 to 10% of all tumors affecting the skin,^2^ and they impose significant morbidity due to treatment modalities that potentially involve the removal of the affected eyelid.

Most reports on the incidence of periocular tumors come from single-center registries. These data are not designed to determine the frequency of new diagnoses in a community or the survival at the population level. The effect of different environmental, social, and economic factors can best be ascertained through population-based studies. Furthermore, cancer registries are the basis for studies that aim to delineate the causes of tumors and to evaluate the efficacy of employed preventive and early detection strategies.

Germany has the largest population in the European Union with 83 million inhabitants as of 2019.^3^ To date, no study has addressed the national incidence and survival rates of patients with malignant tumors of the eyelid at the population level in Germany. The 16 German federated states have been obligated to report epidemiologic cancer data by law since 2009. Through a robust process, data are submitted once a year to the Robert Koch Institute, a disease control and prevention institute subordinate to the Federal Ministry of Health.^4,5^

The aim of this report is to delineate the incidence and survival rates of different malignant tumors of the eyelid in Germany and to examine the different factors that influence these rates at the national level.

## Methods

Cancer is a reportable disease in Germany, and data were provided anonymously to the researchers. Therefore, the study was treated as non-human subject research and was waived from the requirement of signed consent forms. The study adhered to guidelines of good clinical practice, the Declaration of Helsinki, and its amendments.

### Study population and data source

Data were extracted from the German cancer registry operated by the German Center for Cancer Registry Data at the Robert Koch Institute, Berlin. We included data covering the whole population of Germany pertaining to patients 15 years of age or older. For the purposes of conducting population statistics, patient data from all German states reported between 2009 and 2015 were collected. For survival analysis, we included data of all patients reported by the cancer registries for the same period. Data collection and quality control of the national cancer registry have been described before.^5^ Survival time was provided by the Robert Koch Institute as well.

### Coding and data extraction

Patients were selected according to the third edition of the International Classification of Diseases for Oncology (ICD-O-3) topography code C44.1 “Eyelid including canthus”. Associated histology codes were recorded as follows: 8090-8098 as basal cell carcinoma, 8050–8078 and 8083–8084 as squamous cell carcinoma, 8720–8790 as malignant melanoma, 8079–8082, 8099–8576, 8036–8049, and 8085–8089 as other specified carcinomas, and 8010–8035 as unspecified carcinomas.^6^ Specified and unspecified carcinoma were grouped as “other carcinomas”. Histology codes 8800–8811, 8830, 8840–8921, 8990–8991, 9040–9044, 9120–9133, 9150, and 9540–9581 belonged to leukemia, lymphoma, sarcoma and unspecified malignancies, which were grouped as “other malignancies”. TNM staging, method of confirming the diagnosis, and grading were reported. The International Classification of Diseases version 10 (ICD-10) was used to define causes of death.^7^ Data pertaining to patients whose deaths were reported by death certificate only (N = 85) or by autopsy (N = 0) were excluded.

### Statistics

Crude (CR) and Age-standardized (ASR) incidence rates were calculated per year. The 2011 Census population estimates, as reported by the German Federal Statistical Office, were used for the purposes of calculating incidence and standardized incidence rates. The incidence rate was calculated per million due to the small incidence of melanomas. Survival was calculated using the Kaplan-Meier method. For survival analysis, we calculated the *eyelid cancer-specific survival* (ELCSS). Furthermore, we have developed life tables analysis including Wilcoxon (Gehan) Test for group comparisons. The lid-related causes of death were reported as the “event” for calculating cancer-specific survival. COX regression analysis was conducted for the purposes of adjusting for age, sex, and morphology and in order to calculate death-related hazard ratios (HR) and 95% confidence Intervals (95% CI). The Schoenefeld test was used to validate the COX-Model for overall survival.^8^ It was not possible to similarly validate cancer-specific survival using the same test.

The data were organized, cleaned, and the incidence was calculated using Microsoft Excel for Microsoft Office 365.^9^ Descriptive data analysis was conducted using the IBM SPSS version 27.^10^ Incidence Standardization and Confidence Intervals were calculated using PHE Tool.^11^ Maps were plotted using Tableau version 2020.01 software on OpenStreetMaps^12–14^ and R Statistical Packages; “Survival”, “GGPlot2” and “Survminer”.

## Results

### Study population

Data pertaining to 42,710 patients who had been diagnosed with eyelid malignancies were analyzed. A majority of these patients (87.1%; n = 37,181) had been diagnosed with basal cell carcinoma, followed by squamous cell carcinoma (10.1%; n = 4297) and malignant melanoma (1.1%; n = 474). There was a slightly higher overall frequency of malignancies among females (57.4%) as well as higher frequencies of all subtypes except for sarcomas, where male patients made up 75% of the cases (Table 1). Mean age at presentation of all malignancies stood at 69.5 (SD ± 12.76) years of age (Supplementary Table 1). Mean age at diagnosis of basal cell carcinoma stood at 69.38 (SD ± 12.7, 95%CI 69.2–69.5, median 71.58, IQR 17) years of age; whilst that of squamous cell carcinoma stood at 74.6 (SD ± 11.9, 95% CI 74.25–74.97, median 76, IQR 14.58). Mean age at diagnosis of melanoma stood at 72.2 years of age (SD ± 13.4, 95% CI 71.02–73.43, median 75, IQR 14.13). There was a higher frequency of bilateral disease among patients with squamous cell carcinoma (3.8%) compared with other malignancies.

**Table 1.** Patients’ characteristics including sex, state, laterality, and method of the diagnosis.

		Morphology											
		Basal cell carcinoma		Squamous cell carcinoma		Malignant melanoma		Other carcinomas		Other malignancies		Total	
		N	Col N %	N	Col N %	N	Col N %	N	Col N %	N	Col N %	N	Col N %
Sex Chi-Sq p-value <0.0001	Male	15626_a_	42.0%	2050_b_	47.7%	208_a,b_	43.9%	212_a_	40.7%	102_a,b_	43.0%	18,198	42.6%
	Female	21555_a_	58.0%	2247_b_	52.3%	266_a,b_	56.1%	309_a_	59.3%	135_a,b_	57.0%	24,512	57.4%
	Total	37,181	100.0%	4297	100.0%	474	100.0%	521	100.0%	237	100.0%	42,710	100.0%
State p-value <0.0001	Baden-Württemberg	1276_a_	3.4%	134_a_	3.1%	31_b_	6.5%	9_a_	1.7%	24_b_	10.1%	1474	3.5%
	Bayern	3174_a_	8.5%	544_b_	12.7%	99_c_	20.9%	83_b,c_	15.9%	30_a,b,c_	12.7%	3930	9.2%
	Berlin	1205_a_	3.2%	78_b_	1.8%	14_a,b_	3.0%	14_a,b_	2.7%	7_a,b_	3.0%	1318	3.1%
	Brandenburg	434_a_	1.2%	109_b_	2.5%	7_a,b_	1.5%	14_b_	2.7%	7_a,b_	3.0%	571	1.3%
	Bremen	395_a_	1.1%	58_a_	1.3%	4_a_	0.8%	3_a_	0.6%	^1^		460	1.1%
	Hamburg	692_a_	1.9%	74_a_	1.7%	10_a_	2.1%	11_a_	2.1%	3_a_	1.3%	790	1.8%
	Hessen	3113_a,b_	8.4%	356_a,b_	8.3%	50_a_	10.5%	27_b_	5.2%	15_a,b_	6.3%	3561	8.3%
	Mecklenburg-Vorpommern	1192_a_	3.2%	138_a_	3.2%	8_a_	1.7%	10_a_	1.9%	3_a_	1.3%	1351	3.2%
	Niedersachsen	4731_a,b_	12.7%	590_a_	13.7%	41_b,c_	8.6%	39_c_	7.5%	17_b,c,d_	7.2%	5418	12.7%
	Nordrhein-Westfalen	11173_a_	30.1%	1100_b_	25.6%	88_c_	18.6%	196_d_	37.6%	93_d_	39.2%	12,650	29.6%
	Rhineland-Pfalz	2875_a_	7.7%	351_a_	8.2%	34_a_	7.2%	29_a_	5.6%	9_a_	3.8%	3298	7.7%
	Saarland	679_a_	1.8%	68_a_	1.6%	7_a_	1.5%	8_a_	1.5%	^1^		762	1.8%
	Sachsen	2720_a_	7.3%	256_b_	6.0%	27_a,b_	5.7%	30_a,b_	5.8%	10_a,b_	4.2%	3043	7.1%
	Sachsen-Anhalt	508_a_	1.4%	61_a_	1.4%	16_b_	3.4%	9_a,b_	1.7%	4_a,b_	1.7%	598	1.4%
	Schleswig-Holstein	1953_a_	5.3%	259_a_	6.0%	23_a_	4.9%	24_a_	4.6%	12_a_	5.1%	2271	5.3%
	Thüringen	1061_a_	2.9%	121_a_	2.8%	15_a_	3.2%	15_a_	2.9%	3_a_	1.3%	1215	2.8%
	Total	37,181	100.0%	4297	100.0%	474	100.0%	521	100.0%	237	100.0%	42,710	100.0%
Localization p-value <0.0001	Left	15399_a_	41.4%	1766_a_	41.1%	241_b_	50.8%	229_a,b_	44.0%	73_c_	30.8%	17,708	41.5%
	Right	15062_a_	40.5%	1670_a_	38.9%	169_a,b_	35.7%	192_a,b_	36.9%	65_b_	27.4%	17,158	40.2%
	Bilateral	1091_a_	2.9%	162_b_	3.8%	8_a,b_	1.7%	15_a,b_	2.9%	5_a,b_	2.1%	1281	3.0%
	Central	7_a_	0.0%	0^1^	0.0%	0^1^	0.0%	0^1^	0.0%	0^1^	0.0%	7	0.0%
	Not reported	5622_a_	15.1%	699_a_	16.3%	56_a_	11.8%	85_a_	16.3%	94_b_	39.7%	6556	15.4%
	Total	37,181	100.0%	4297	100.0%	474	100.0%	521	100.0%	237	100.0%	42,710	100.0%
Method of confirming diagnosis p-value <0.0001	Clinical diagnosis	169_a_	0.4%	13_a_	0.3%	2_a_	0.4%	1_a_	0.2%	16_b_	6.7%	201	0.4%
	Cytology	72_a_	0.2%	9_a_	0.2%	1_a_	0.2%	^1^		2_a_	0.8%	84	0.2%
	Histology of Metastasis	2_a_	0.0%	^1^		^1^		^1^		^1^		2	0.0%
	Histology of Primary Tumor	36936_a_	99.3%	4275_a_	99.5%	467_a_	98.5%	520_a_	99.8%	219_b_	92.4%	42,417	99.3%
	Others	2_a_	0.0%	^1^		4_b_	0.8%	^1^		^1^		6	0.0%
	Total	37,181	100.0%	4297	100.0%	474	100.0%	521	100.0%	237	100.0%	42,710	100.0%

Note: Values in the same row and subtable not sharing the same subscript are significantly different at p < .05 in the two-sided test of equality for column proportions. Cells with no subscript are not included in the test. Tests assume equal variances.^2^

1. This category is not used in comparisons because its column proportion is equal to zero or one.

2. Tests are adjusted for all pairwise comparisons within a row of each innermost subtable using the Bonferroni correction.

### Incidence

**Figure 1.**
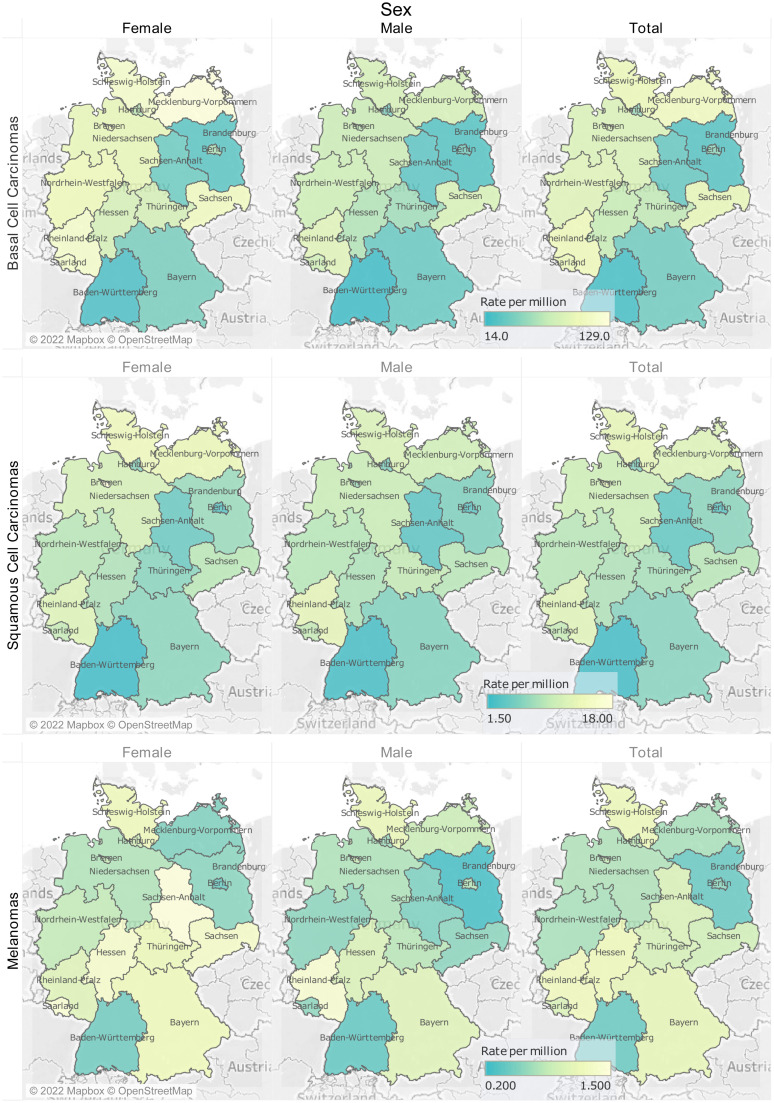
Crude incidence rate of eyelid malignant tumors per state.

**Figure 2.**
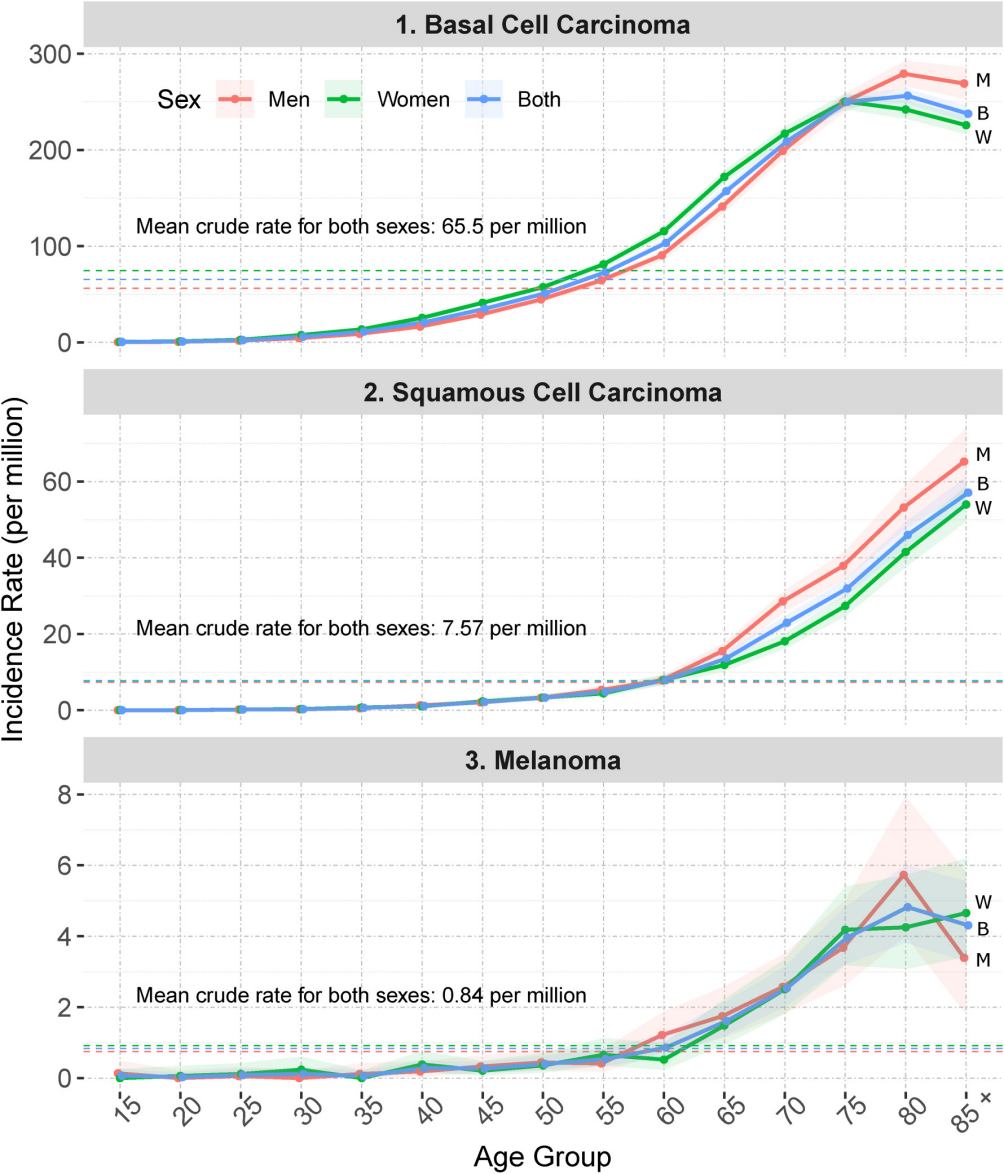
Age-specific incidence rates.

The nationwide ASR for basal cell carcinoma stood at 65.1 per million. The state of Mecklenburg-Vorpommern had the highest CIR for basal cell carcinoma (106 per million). Schleswig-Holstein had the highest CIR (13.1) for squamous cell carcinoma, while Rheinland-Pfalz had the highest CIR for melanoma (1.2) (Figure 1, Supplementary Table 2). The ASR in males stood a lower 55.6 per million compared with the 74.43 in females (rate ratio = 0.7503, p <0.0001). For males, the ASR peaked at 11.5 per million in the 70–74 age group; females in the same age group had an ASR of 13.9 per million (Figure 2, Supplementary Table 3). Whilst the ASR in males had peaked at 70–74 years of age, the ASR in females continued to rise until a peak at over 85 years of age. The ASR of melanoma stood at 0.83 per million, with a rate of 0.74 in males and 0.91 in females (rate ratio 0.81, p-value < 0.0001) and a peak of 0.15 in the 70–74 age group in males and 0.19 in the 75–79 age in females. It is worth mentioning that CIR for females was higher than that of males, whilst the higher ASR in females reflected their nationwide population distribution. Analysis revealed no clearly discernable temporal or geographical trends over the study period (Figure 1, Supplementary Figure 1). Out of the 42,710 incidences, one patient showed two consequent lid malignancies, 140 patients showed single second lid malignancy. Squamous cell carcinomas following basal cell carcinoma were the most common second malignancy (N = 82).

### Diagnosis, histopathology and treatment

Histological confirmation was the most common method of diagnosis (99.3%). Pathological TNM was reported to the registry in 97.5% of the TNM prefix (pTNM). Out of 17,349 patients with reported TNM-T, T1 was the most common stage (N = 12,143, 70.0%), followed by unclear stage Tx (N = 3347, (19.3%) (Table 2). Out of 6458 with a reported grade of differentiation, 63.5% (N = 4102) of patients had presented with well-differentiated (G-1) tumors. Regional and distant metastatic tumors were uncommon (<1%). Surgery, as the sole modality of treatment was conducted in 88% of cases with basal cell carcinoma, 81% of cases with squamous cell carcinomas, and 73% of cases with malignant melanomas (Table 3).

**Table 2.** Pathological characteristics, and staging of the tumors.

		Morphology											
		Basal cell carcinoma		Squamous cell carcinoma		Malignant melanoma		Other carcinomas		Other malignancies		Total	
		N	Col N %	N	Col N %	N	Col N %	N	Col N %	N	Col N %	N	Col N %
Prefix T p-value <0.0001	Pretherapeutic Clinical	172_a_	2.3%	51_a_	2.8%	6_a_	2.3%	7_a_	3.9%	4_b_	23.5%	240	2.5%
	Postoperative Histopathological	7207_a_	97.7%	1770_a_	97.2%	257_a_	97.7%	171_a_	96.1%	13_b_	76.5%	9418	97.5%
	Total	7379	100.0%	1821	100.0%	263	100.0%	178	100.0%	17	100.0%	9658	100.0%
TNM T p-value <0.0001	0 (main cannot be found)	17_a_	0.1%	2_a_	0.1%	^1^		^1^		^1^		19	0.1%
	1	9575_a_	69.2%	2214_b_	77.0%	209_c_	57.1%	133_c_	55.0%	12_c_	37.5%	12,143	70.0%
	2	1065_a_	7.7%	353_b_	12.3%	55_b_	15.0%	66_c_	27.3%	1_a,b_	3.1%	1540	8.9%
	3	68_a_	0.5%	97_b_	3.4%	54_c_	14.8%	15_b_	6.2%	1_a,b,c_	3.1%	235	1.4%
	4	8_a_	0.1%	10_b_	0.3%	38_c_	10.4%	7_d_	2.9%	^1^		63	0.4%
	is	^1^		2_a_	0.1%	^1^		^1^		^1^		2	0.0%
	X (Main tumor cannot be measured)	3102_a_	22.4%	196_b_	6.8%	10_c_	2.7%	21_b_	8.7%	18_d_	56.3%	3347	19.3%
	Total	13,835	100.0%	2874	100.0%	366	100.0%	242	100.0%	32	100.0%	17,349	100.0%
Prefix N p-value <0.0001	Pretherapeutic Clinical	3112_a_	76.2%	709_a_	75.2%	106_b_	61.6%	57_b_	60.0%	7_a,b_	87.5%	3991	75.2%
	Postoperative Histopathological	974_a_	23.8%	234_a_	24.8%	66_b_	38.4%	38_b_	40.0%	1_a,b_	12.5%	1313	24.8%
	Total	4086	100.0%	943	100.0%	172	100.0%	95	100.0%	8	100.0%	5304	100.0%
TNM N p-value <0.0001	0	6514_a_	61.3%	1226_a_	64.2%	222_b_	80.4%	105_a_	65.6%	7_c_	28.0%	8074	62.1%
	1	3_a_	0.0%	26_b_	1.4%	2_b_	0.7%	9_c_	5.6%	^1^		40	0.3%
	2	^1^		1_a_	0.1%	2_b_	0.7%	2_b_	1.3%	^1^		5	0.0%
	3	^1^		^1^		^1^		^1^		^1^			
	x	4116_a_	38.7%	658_b_	34.4%	50_c_	18.1%	44_b,c_	27.5%	18_d_	72.0%	4886	37.6%
	Total	10,633	100.0%	1911	100.0%	276	100.0%	160	100.0%	25	100.0%	13,005	100.0%
Prefix M p-value .283	Pretherapeutic Clinical	5256_a_	87.5%	1046_a_	89.5%	180_a_	88.2%	127_a_	87.6%	9^1^	100.0%	6618	87.8%
	Postoperative Histopathological	754_a_	12.5%	123_a_	10.5%	24_a_	11.8%	18_a_	12.4%	^1^		919	12.2%
	Total	6010	100.0%	1169	100.0%	204	100.0%	145	100.0%	9	100.0%	7537	100.0%
TNM M p-value <0.0001	0	7984_a_	68.4%	1473_b_	77.9%	255_c_	90.4%	164_b,c_	85.9%	8_d_	32.0%	9884	70.3%
	1	3_a_	0.0%	6_b_	0.3%	8_c_	2.8%	^1^		^1^		17	0.1%
	x	3677_a_	31.5%	412_b_	21.8%	19_c_	6.7%	27_b,c_	14.1%	17_d_	68.0%	4152	29.5%
	Total	11,664	100.0%	1891	100.0%	282	100.0%	191	100.0%	25	100.0%	14,053	100.0%
Grading p-value <0.0001	Well differentiated	2453_a_	84.5%	1608_b_	48.0%	1_b,c_	11.1%	35_c_	19.3%	5_b,c_	35.7%	4102	63.5%
	Moderately differentiated	304_a_	10.5%	1386_b_	41.4%	^1^		71_b_	39.2%	2_a,b_	14.3%	1763	27.3%
	Poorly differentiated	98_a_	3.4%	333_b_	9.9%	6_c_	66.7%	67_c,d_	37.0%	1_a,b,d_	7.1%	505	7.8%
	Undifferentiated	45_a_	1.5%	14_b_	0.4%	2_c_	22.2%	6_a_	3.3%	^1^		67	1.0%
	Total	2900	100.0%	3341	100.0%	9	100.0%	179	100.0%	6	100.0%	6437	100.0%

Note: Values in the same row and subtable not sharing the same subscript are significantly different at p < .05 in the two-sided test of equality for column proportions. Cells with no subscript are not included in the test. Tests assume equal variances.^2^

1. This category is not used in comparisons because its column proportion is equal to zero or one.

2. Tests are adjusted for all pairwise comparisons within a row of each innermost subtable using the Bonferroni correction.

**Table 3.** Treatment of the patients and final follow-up status.

		Morphology											
		Basal cell carcinoma		Squamous cell carcinoma		Malignant melanoma		Other carcinomas		Other malignancies		Total	
		N	Col N %	N	Col N %	N	Col N %	N	Col N %	N	Col N %	N	Col N %
Treatment p-value <.0001	Surgery, Radiotherapy and Chemotherapy	^1^		2_a_	0.6%	^1^		1_a_	1.6%	^1^		3	0.1%
	Surgery, Chemotherapy, and others	1_a_	0.0%	1_a,b_	0.3%	^1^		2_b_	3.3%	^1^		4	0.1%
	Surgery and Radiotherapy	16_a_	0.6%	^1^		^1^		10_b_	16.4%	3_b_	14.3%	29	0.9%
	Surgery, Chemotherapy and Others	^1^		^1^		1_a_	2.4%	^1^		2_a_	9.5%	3	0.1%
	Surgery and Chemotherapy	3_a_	0.1%	3_b_	1.0%	^1^		^1^		^1^		6	0.2%
	Surgery and Others	196_a_	7.3%	29_a,b_	9.3%	9_b_	22.0%	4_a,b_	6.6%	1_a,b_	4.8%	239	7.7%
	Only Surgery	2356_a_	88.1%	253_b_	80.8%	30_b,c_	73.2%	40_b,c_	65.6%	11_c_	52.4%	2690	86.5%
	Only Radiotherapy	8_a_	0.3%	1_a,b_	0.3%	^1^		2_b,c_	3.3%	2_c_	9.5%	13	0.4%
	Only Chemotherapy	1_a_	0.0%	^1^		^1^		^1^		^1^		1	0.0%
	Only others	16_a_	0.6%	8_b_	2.6%	^1^		^1^		1_b_	4.8%	25	0.8%
	None	77_a_	2.9%	16_a_	5.1%	1_a_	2.4%	2_a_	3.3%	1_a_	4.8%	97	3.1%
	Total	2674	100.0%	313	100.0%	41	100.0%	61	100.0%	21	100.0%	3110	100.0%
Survival	Dead	5240_a_	14.1%	1122_b_	26.1%	130_b,c_	27.4%	175_c_	33.6%	57_b,c_	24.1%	6724	15.7%
	Alive	31941_a_	85.9%	3175_b_	73.9%	344_b,c_	72.6%	346_c_	66.4%	180_b,c_	75.9%	35986	84.3%
	Total	37,181	100.0%	4297	100.0%	474	100.0%	521	100.0%	237	100.0%	42,710	100.0%

Note: Values in the same row and subtable not sharing the same subscript are significantly different at p < .05 in the two-sided test of equality for column proportions. Cells with no subscript are not included in the test. Tests assume equal variances.^2^

1. This category is not used in comparisons because its column proportion is equal to zero or one.

2. Tests are adjusted for all pairwise comparisons within a row of each innermost subtable using the Bonferroni correction.

### Survival

**Figure 3.**
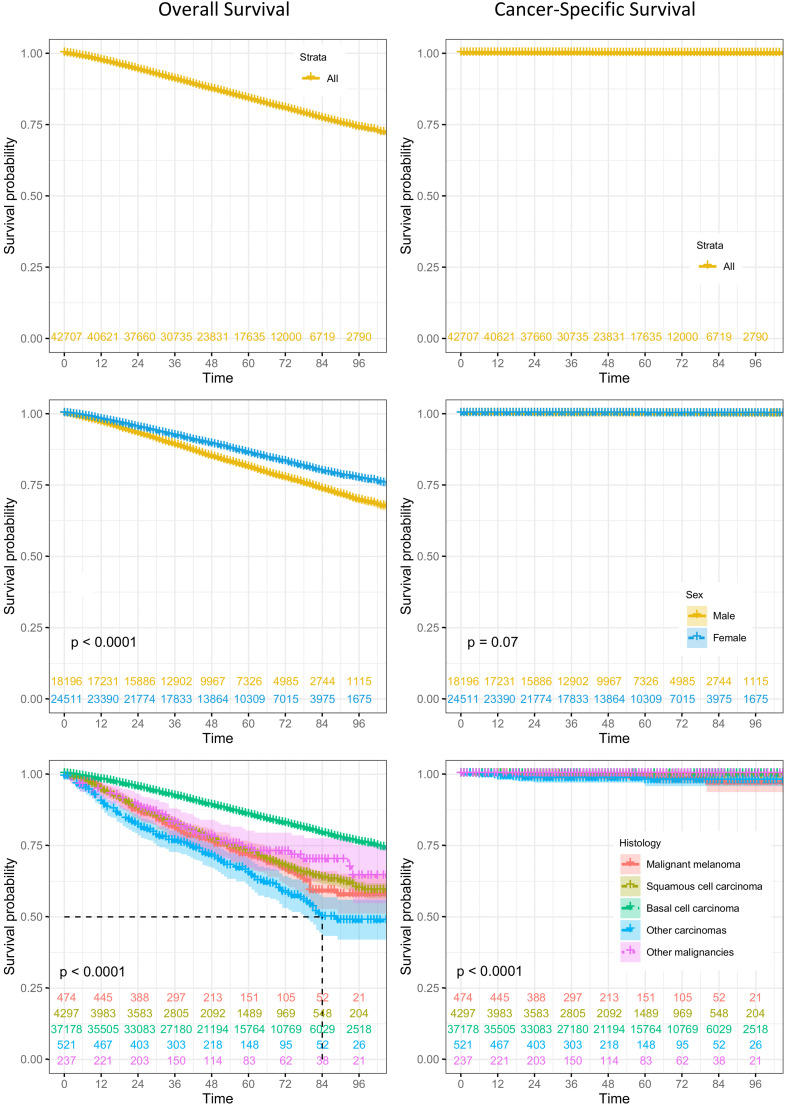
Overall survival (left column) and cancer-specific survival (right column) by age, gender and morphology.

All patients had ELCSS above 95%. Patients with squamous cell carcinoma had the worst ELCSS (Figure 3). Patients with malignant melanomas had the lowest survival rate (96.4%). Patients with squamous cell carcinoma and patients with basal cell carcinoma showed higher survival of 99.5%, and 99.9%, respectively (Figure 3, Supplementary Table 4).

Overall survival at five years after a diagnosis of squamous cell carcinoma and malignant melanoma stood at comparable rates of 58.3% and 54.8%, respectively. Both rates were considerably lower than the overall survival rate at five years after a diagnosis of basal cell carcinoma, which stood at 74.9% (Figure 3, Supplementary Table 5). Overall, males had lower overall survival probabilities at five years after their diagnoses compared with females.

#### COX regression survival

**Figure 4.**
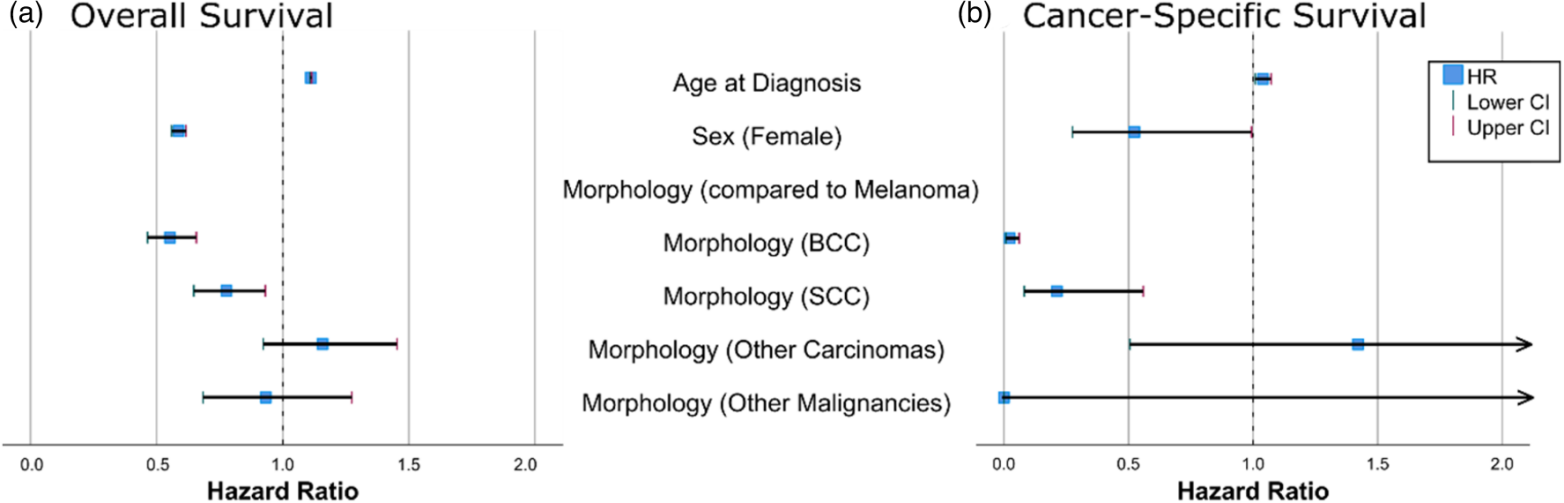
Cox regression analysis showing hazard ratio for overall (a) and cancer specific survival (b).

Multivariate analysis revealed that male patients had lower overall survival rates (HR = 1.7, 95% CI 1.62–1.79). Moreover, the older the age at diagnosis, the higher the risk of death (HR = 1.11, 95%CI 1.11- 1.12). Patients diagnosed with basal cell carcinoma and squamous cell carcinoma had the best overall survival. Evidently, due to their shared confidence intervals, the survival rates of patients with basal cell and squamous cell carcinomas did not significantly differ. The same patterns were noted after a multivariate analysis of cancer-specific survival. Further details are presented in Figure 4, Supplementary Tables 6–7, and Supplementary Figure 2.

## Discussion

The current study is the first to systematically report on the incidence of malignant tumors of the eyelid in Germany. This was possible primarily because all German states have been obligated to report their cancer data to the Robert Koch Institute since 2009. Until now, published data have only been available for “skin tumors in general”, with non-melanoma ASRs of 184 and 143 per 100.000 for males and females, respectively.^15^ As there are geographic factors in the whole of Europe, which are evident within Germany, a separate analysis of epidemiologic data of the individual German states, conducted in a similar manner to the nationwide analysis conducted in this study, should be undertaken.^16^

An overview of available eyelid tumor population-based studies from other countries is found in Supplementary Table 8. As evident by the overview, a heterogeneous set of conceptions have stood behind the buildup of these studies. The only European study comparable to this work was an Irish study with an observation period between 2005 and 2015. It reported ASRs for patients with basal cell carcinoma of 158.7 and 134.9 per million for males and females, respectively,^17^ which were about twice as high as our rates. Compared to the Irish study’s reported squamous cell carcinoma ASRs of 21.0 (males) and 13.9 (females), the same ASRs stood at 7.25 (males) and 7.7 (females) in our study. The literature review revealed that basal cell carcinoma was the most common lid malignancy, ranging between 80–95%.^18^ It followed the age distribution of skin basal cell carcinomas described before; however, our study showed more precision. Although melanomas represented a considerably small proportion of malignancies reported by this and other comparable studies, it continues to carry a significant burden of clinical morbidity and mortality.

Interestingly, sebaceous gland carcinoma was reportedly the most frequent eyelid malignancy in India,^19^ the second most in South Korea and Singapore,^20,21^ and the third most frequent in Taiwan.^22^ In our study, sebaceous gland carcinoma was found to be a rare malignancy (N = 135). This finding is suggestive of strong geographic and, possibly, genetic variations responsible for these differences.

In males, there was a steady increase in the incidences of the three most common malignancies, which peaked at the 70–75 age group, then a decline afterwards. In females, the incidence of squamous cell carcinoma continued to rise beyond the 70–75 age group. This is possibly attributable to the accumulation of genetic mutations with aging and infections with a higher cumulative exposure to ultraviolet light as well as reduced repair and a higher apoptosis capacity.^23,24^ We postulate that the decline in the overall incidence of malignancies in older age groups may reflect a decreased interest in seeking medical attention in these groups (e.g., due to lack of interest in improving one’s body figure). This may in turn explain the lower survival probabilities among the very old, especially among male patients.^25^

Previous studies have reported an association between higher rates of squamous cell carcinomas and the male gender.^26,17^ These results were comparable to those of our study in males up to the age of 75. Beyond the age of 75, females had the higher ASRs. In the Irish study, higher ASRs of basal cell carcinoma were reported among males. Conversely, ASRs were higher among females in this study.

Previous studies reported rising incidences of basal cell carcinoma and melanoma of the skin over long periods.^1,27–29^ As the data collected for the purposes of this study cover only a relatively short period, no conclusions about long term trend could be drawn. Earlier studies of eyelid malignant tumors reported non-significant trend of rising incidences.^17,30^ Further studies with more extended observation periods are needed in order to better understand the significance of this trend.

Survival rates in malignant tumors of the eyelid have rarely been reported. As with this work, Jung et al. reported a near 100% survival of patients with BCC.^20^ The cancer-specific survival of skin tumors is high and has been continuously rising over the years.^31–33^ Higher mortality rates have been reported for sebaceous gland carcinoma^34^ and melanoma.^35^

Although a considerable effort was made in data collection, quality control, and analysis, there might be some limitations to our study. The study’s retrospective registry-based design renders it prone to the bias of incomplete data. A known similar example is found in the Surveillance, Epidemiology, and End-Results (SEER) program, where the data on treatment may not reflect the latest status of patients. However, in SEER, roughly one-third of the US population is included, whereas our study comprises the whole of Germany.

In this study, differences between the German states and other European countries with regards to the incidences of tumors may be attributable to a set of factors. As solar radiation is essentially equally distributed over northwestern Europe^36^ and also within Germany, it seems unlikely to be a factor in these differences. Socioeconomic differences might be playing a role, as it has been shown that the incidence of cancer is unequally distributed across socioeconomic groups in Germany.^37^ Differences in skin type and genetic factors have not been assessed. Furthermore, an essential influencing factor may be the reporting system itself, which is theoretically homogonous, but, in practice, heterogeneous. The discrepancy in medical coverage (or its accessibility) and different levels of interest in seeking treatment are possible confounders. This is thus a possible disadvantage of conducting register data-based studies comparted to retrospective case series.

Care needed to be taken when data of different German states were added together, as the respective registers had varied levels of completeness.^15^ States declared explicitly as reference regions by the Robert Koch Institute are Saarland, Hamburg, Schleswig-Holstein, Niedersachsen, as well as some regions in Bavaria and Nordrhein-Westfalen, each having been reporting area-wide data for at least ten years.^15^ A law obligating federal states to collect clinical cancer data from 2013 is still in the phase of implementation. Thus, robust data concerning therapy and the course of cancer illness are not currently available in the whole of Germany and could therefore not be presented here. Moreover, our study did not examine the competing causes of death which can be an area for future research.

Actual numbers could be higher than the reported ones here because relapses and multiple tumors were not being recorded by the registries. Apart from that, there might be bias in the data due to the waiving of invasive diagnostic procedures in the very old, resulting in a possible underestimation of incidences in the highest age group.^27^ Future numbers might significantly change upwards due to the aging German society or downwards due to the immigration of younger people of non-Caucasian descent who may have lower risks of non-melanocytic skin tumors. Numbers may also rise through the implementation of effective of early detection measures.

In summary, this is the first study to present data pertaining to the incidences of eyelid malignances in Germany. Our results and findings may provide a basis for the improvement of cancer registration and health education concerning eyelid malignances. Future studies might be able to better identify high-risk groups and recommend the best prevention strategies.

## Supplemental Material

sj-docx-1-ejo-10.1177_11206721221125018 - Supplemental material for The epidemiology of adults' eyelid malignancies in Germany between 2009 and 2015; An analysis of 42,710 patients' data Click here for additional data file. Supplemental material, sj-docx-1-ejo-10.1177_11206721221125018 for The epidemiology of adults' eyelid malignancies in Germany between 2009 and 2015; An analysis of 42,710 patients' data by Ahmad S Alfaar, C Nathanael Suckert, Matus Rehak and Christian Girbardt in European Journal of Ophthalmology

sj-xlsx-2-ejo-10.1177_11206721221125018 - Supplemental material for The epidemiology of adults' eyelid malignancies in Germany between 2009 and 2015; An analysis of 42,710 patients' data Click here for additional data file. Supplemental material, sj-xlsx-2-ejo-10.1177_11206721221125018 for The epidemiology of adults' eyelid malignancies in Germany between 2009 and 2015; An analysis of 42,710 patients' data by Ahmad S Alfaar, C Nathanael Suckert, Matus Rehak and Christian Girbardt in European Journal of Ophthalmology

sj-xlsx-3-ejo-10.1177_11206721221125018 - Supplemental material for The epidemiology of adults' eyelid malignancies in Germany between 2009 and 2015; An analysis of 42,710 patients' data Click here for additional data file. Supplemental material, sj-xlsx-3-ejo-10.1177_11206721221125018 for The epidemiology of adults' eyelid malignancies in Germany between 2009 and 2015; An analysis of 42,710 patients' data by Ahmad S Alfaar, C Nathanael Suckert, Matus Rehak and Christian Girbardt in European Journal of Ophthalmology
