# Supplementary material for: The epidemiology of adults' eyelid malignancies in Germany between 2009 and 2015; An analysis of 42,710 patients' data
Source: Eur J Ophthalmol. 2022 Nov 4;33(2):1186–99. doi: 10.1177/11206721221125018 (PMC9999282; doi:10.1177/11206721221125018)

Supplementary Tables:

Supplementary table 1: Age at presentation in years.

| Morphology | Squamous cell carcinoma | Basal cell carcinoma | Malignant melanoma | Other carcinomas | Other malignancies |
| --- | --- | --- | --- | --- | --- |
| Mean | 74.6 | 69.4 | 72.2 | 74.5 | 69.2 |
| Mean SE | 0.2 | 0.1 | 0.6 | 0.5 | 0.9 |
| 95% CI Mean Upper | 74.3 | 69.2 | 71.0 | 73.5 | 67.4 |
| 95% CI Mean lower | 75.0 | 69.5 | 73.4 | 75.6 | 71.1 |
| 5% Trimmed Mean | 75.2 | 69.8 | 73.1 | 75.2 | 69.8 |
| Median | 76.0 | 71.6 | 75.0 | 76.5 | 71.3 |
| Variance | 141.8 | 161.7 | 179.5 | 146.5 | 211.5 |
| Std. Deviation | 11.9 | 12.7 | 13.4 | 12.1 | 14.5 |
| Minimum | 27.1 | 15.3 | 15.3 | 15.0 | 17.7 |
| Maximum | 104.0 | 115.5 | 100.3 | 101.5 | 97.2 |
| Range | 76.9 | 100.2 | 85.0 | 86.5 | 79.5 |
| Interquartile Range | 14.6 | 17.0 | 14.1 | 14.7 | 18.7 |
| Skewness | -0.8 | -0.6 | -1.2 | -1.0 | -0.7 |
| Skewness SE | 0.0 |  | 0.1 | 0.1 | 0.2 |
| Kurtosis | 0.6 | 0.1 | 1.8 | 1.9 | 0.3 |
| Kurtosis SE | 0.1 | 0.0 | 0.2 | 0.2 | 0.3 |

Supplementary Data 2: Age-standardized incidence rates (ASIR) and number of patients for Basal cell carcinoma (BCC), Squamous cell carcinoma (SCC) and Melanoma (Mel).

- *Excel Sheet*

Supplementary Data 3: Crude incidence rate per state for each malignancy.

- *Excel Sheet*

Supplementary table 4.a: Overall survival – lifetable.

|  | Interval Start Time | Number Entering Interval | Number Withdrawing during Interval | Number of Terminal Events | Cumulative Proportion Surviving at End of Interval |
| --- | --- | --- | --- | --- | --- |
| Malignant melanoma | 0 | 474 | 15 | 30 | 0.94 |
|  | 15 | 429 | 40 | 36 | 0.85 |
|  | 30 | 353 | 88 | 30 | 0.77 |
|  | 45 | 235 | 70 | 14 | 0.72 |
|  | 60 | 151 | 131 | 20 | 0.55 |
| Squamous cell carcinoma | 0 | 4297 | 135 | 280 | 0.93 |
|  | 15 | 3882 | 365 | 305 | 0.86 |
|  | 30 | 3212 | 728 | 227 | 0.79 |
|  | 45 | 2257 | 625 | 143 | 0.73 |
|  | 60 | 1489 | 1322 | 167 | 0.58 |
| Basal cell carcinoma | 0 | 37178 | 1375 | 882 | 0.98 |
|  | 15 | 34921 | 3535 | 1251 | 0.94 |
|  | 30 | 30135 | 6321 | 1153 | 0.90 |
|  | 45 | 22661 | 6020 | 877 | 0.86 |
|  | 60 | 15764 | 14687 | 1077 | 0.75 |
| Other specified carcinomas | 0 | 469 | 15 | 57 | 0.88 |
|  | 15 | 397 | 41 | 40 | 0.78 |
|  | 30 | 316 | 76 | 20 | 0.73 |
|  | 45 | 220 | 71 | 18 | 0.66 |
|  | 60 | 131 | 106 | 25 | 0.45 |
| Unspecified carcinoma | 0 | 52 | 3 | 4 | 0.92 |
|  | 15 | 45 | 2 | 3 | 0.86 |
|  | 30 | 40 | 13 | 3 | 0.78 |
|  | 45 | 24 | 4 | 3 | 0.67 |
|  | 60 | 17 | 15 | 2 | 0.53 |
| Sarcoma | 0 | 24 | 1 | 0 | 1.00 |
|  | 15 | 23 | 4 | 0 | 1.00 |
|  | 30 | 19 | 1 | 2 | 0.89 |
|  | 45 | 16 | 5 | 0 | 0.89 |
|  | 60 | 11 | 9 | 2 | 0.62 |
| Unspecified malignant neoplasm | 0 | 94 | 0 | 6 | 0.94 |
|  | 15 | 88 | 11 | 2 | 0.91 |
|  | 30 | 75 | 21 | 4 | 0.86 |
|  | 45 | 50 | 15 | 2 | 0.82 |
|  | 60 | 33 | 32 | 1 | 0.77 |
| Lymphomas | 0 | 118 | 2 | 9 | 0.92 |
|  | 15 | 107 | 13 | 13 | 0.80 |
|  | 30 | 81 | 12 | 9 | 0.71 |
|  | 45 | 60 | 17 | 4 | 0.65 |
|  | 60 | 39 | 37 | 2 | 0.59 |

Supplementary table 4.b: Significance of cross-comparisons using Wilcoxon (Gehan) statistic in overall survival lifetable

|  | Squamous cell carcinoma | Basal cell carcinoma | Other specified carcinomas | Unspecified carcinoma | Sarcoma | Unspecified malignant neoplasm | Lymphomas |  |
| --- | --- | --- | --- | --- | --- | --- | --- | --- |
| Malignant melanoma | 0.412 | 87.064 | 7.132 | 0.000 | 3.282 | 3.483 |  | Statistic |
|  | 0.521 | 0.000 | 0.008 | 0.994 | 0.070 | 0.062 |  | Sig. |
| Squamous cell carcinoma |  | 534.059 | 18.805 | 0.069 | 2.846 | 2.971 | 2.372 | Statistic |
|  |  | 0.000 | 0.000 | 0.793 | 0.092 | 0.085 | 0.124 | Sig. |
| Basal cell carcinoma |  |  | 203.769 | 10.315 | 0.088 | 1.620 | 40.673 | Statistic |
|  |  |  | 0.000 | 0.001 | 0.766 | 0.203 | 0.000 | Sig. |
| Other specified carcinomas |  |  |  | 1.221 | 5.781 | 9.530 | 0.449 | Statistic |
|  |  |  |  | 0.269 | 0.016 | 0.002 | 0.503 | Sig. |
| Unspecified carcinoma |  |  |  |  | 3.016 | 1.751 | 0.354 | Statistic |
|  |  |  |  |  | 0.082 | 0.186 | 0.552 | Sig. |
| Sarcoma |  |  |  |  |  | 0.688 | 4.351 | Statistic |
|  |  |  |  |  |  | 0.407 | 0.037 | Sig. |
| Unspecified malignant neoplasm |  |  |  |  |  |  | 5.317 | Statistic |
|  |  |  |  |  |  |  | 0.021 | Sig. |

Supplementary table 5.a: Cancer-specific survival – lifetable

|  | Interval Start Time | Number Entering Interval | Number Withdrawing during Interval | Number of Terminal Events | **Cumulative Proportion Surviving at End of Interval** |
| --- | --- | --- | --- | --- | --- |
| Malignant melanoma | 0 | 474 | 45 | 0 | **1.000** |
|  | 15 | 429 | 72 | 4 | **0.990** |
|  | 30 | 353 | 118 | 0 | **0.990** |
|  | 45 | 235 | 84 | 0 | **0.990** |
|  | 60 | 151 | 149 | 2 | **0.964** |
| Squamous cell carcinoma | 0 | 4297 | 411 | 4 | **0.999** |
|  | 15 | 3882 | 667 | 3 | **0.998** |
|  | 30 | 3212 | 951 | 4 | **0.997** |
|  | 45 | 2257 | 767 | 1 | **0.996** |
|  | 60 | 1489 | 1488 | 1 | **0.995** |
| Basal cell carcinoma | 0 | 37178 | 2256 | 1 | **1.000** |
|  | 15 | 34921 | 4784 | 2 | **1.000** |
|  | 30 | 30135 | 7471 | 3 | **1.000** |
|  | 45 | 22661 | 6896 | 1 | **1.000** |
|  | 60 | 15764 | 15760 | 4 | **0.999** |
| Other specified carcinoma | 0 | 469 | 67 | 5 | **0.989** |
|  | 15 | 397 | 79 | 2 | **0.983** |
|  | 30 | 316 | 96 | 0 | **0.983** |
|  | 45 | 220 | 89 | 0 | **0.983** |
|  | 60 | 131 | 130 | 1 | **0.968** |
| Unspecified carcinoma | 0 | 52 | 7 | 0 | **1.000** |
|  | 15 | 45 | 4 | 1 | **0.977** |
|  | 30 | 40 | 16 | 0 | **0.977** |
|  | 45 | 24 | 7 | 0 | **0.977** |
|  | 60 | 17 | 17 | 0 | **0.977** |
| Sarcoma | 0 | 24 | 1 | 0 | **1.000** |
|  | 15 | 23 | 4 | 0 | **1.000** |
|  | 30 | 19 | 3 | 0 | **1.000** |
|  | 45 | 16 | 5 | 0 | **1.000** |
|  | 60 | 11 | 11 | 0 | **1.000** |
| Unspecified malignant neoplasm | 0 | 94 | 6 | 0 | **1.000** |
|  | 15 | 88 | 13 | 0 | **1.000** |
|  | 30 | 75 | 25 | 0 | **1.000** |
|  | 45 | 50 | 17 | 0 | **1.000** |
|  | 60 | 33 | 33 | 0 | **1.000** |
| Lymphomas | 0 | 118 | 11 | 0 | **1.000** |
|  | 15 | 107 | 26 | 0 | **1.000** |
|  | 30 | 81 | 21 | 0 | **1.000** |
|  | 45 | 60 | 21 | 0 | **1.000** |
|  | 60 | 39 | 39 | 0 | **1.000** |

Supplementary table 5.b: Significance of cross-comparisons using Wilcoxon (Gehan) statistic in Cancer-specific survival lifetable

|  | Squamous cell carcinoma | Basal cell carcinoma | Other specified carcinoma | Unspecified carcinoma | Sarcoma | Unspecified malignant neoplasm | Lymphomas |  |
| --- | --- | --- | --- | --- | --- | --- | --- | --- |
| Malignant melanoma | 7.512 | 165.400 | 1.202 | 0.437 | 0.316 | 1.089 | 1.230 | Statistic |
|  | 0.006 | 0.000 | 0.273 | 0.509 | 0.574 | 0.297 | 0.267 | Sig. |
| Squamous cell carcinoma |  | 58.563 | 23.646 | 5.797 | 0.082 | 0.279 | 0.304 | Statistic |
|  |  | 0.000 | 0.000 | 0.016 | 0.775 | 0.597 | 0.582 | Sig. |
| Basal cell carcinoma |  |  | 345.464 | 104.745 | 0.007 | 0.020 | 0.023 | Statistic |
|  |  |  | 0.000 | 0.000 | 0.933 | 0.886 | 0.880 | Sig. |
| Other specified carcinoma |  |  |  | 0.004 | 0.472 | 1.724 | 2.039 | Statistic |
|  |  |  |  | 0.951 | 0.492 | 0.189 | 0.153 | Sig. |
| Unspecified carcinoma |  |  |  |  | 0.500 | 1.910 | 2.256 | Statistic |
|  |  |  |  |  | 0.480 | 0.167 | 0.133 | Sig. |

Supplementary table 6: Cox Regression Model Details for Overall Survival

| **Omnibus Tests of Model Coefficients^a^** | | | | | | | | | |
| --- | --- | --- | --- | --- | --- | --- | --- | --- | --- |
| -2 Log Likelihood | Overall (score) | | | Change From Previous Step | | | Change From Previous Block | | |
|  | Chi-square | df | Sig. | Chi-square | df | Sig. | Chi-square | df | Sig. |
| 130958.157 | 5763.379 | 6 | .0001 | 7113.476 | 6 | .0001 | 7113.476 | 6 | .0001 |
| a. Beginning Block Number 1. Method = Enter | | | | | | | | | |

**Variables in the Equation**

|  | Sig. | Exp(B) | 95.0% CI for Exp(B) | |
| --- | --- | --- | --- | --- |
|  |  |  | Lower | Upper |
| Sex | <.0001 | .597 | .568 | .626 |
| Age at Diagnosis | <.0001 | 1.108 | 1.105 | 1.111 |
| Morphology | <.0001 |  |  |  |
| Morphology (SCC) | .006 | .775 | .647 | .930 |
| Morphology (BCC) | <.0001 | .562 | .472 | .668 |
| Morphology (Other Carcinomas) | .275 | 1.135 | .904 | 1.424 |
| Morphology (Other malignancies) | .677 | .936 | .686 | 1.278 |

Supplementary table 7: Cox Regression Model Details for Cancer-Specific Survival

| **Omnibus Tests of Model Coefficients^a^** | | | | | | | | | |
| --- | --- | --- | --- | --- | --- | --- | --- | --- | --- |
| -2 Log Likelihood | Overall (score) | | | Change From Previous Step | | | Change From Previous Block | | |
|  | Chi-square | df | Sig. | Chi-square | df | Sig. | Chi-square | df | Sig. |
| 702.415 | 306.513 | 6 | .0001 | 104.992 | 6 | .0001 | 104.992 | 6 | .0001 |
| a. Beginning Block Number 1. Method = Enter | | | | | | | | | |

**Variables in the Equation**

|  | Sig. | Exp(B) | 95.0% CI for Exp(B) | |
| --- | --- | --- | --- | --- |
|  |  |  | Lower | Upper |
| Sex | .054 | .532 | .280 | 1.010 |
| Age at Diagnosis | .021 | 1.037 | 1.005 | 1.069 |
| Morphology | .000 |  |  |  |
| Morphology (SCC) | .002 | .214 | .081 | .564 |
| Morphology (BCC) | .000 | .023 | .008 | .062 |
| Morphology (Other Carcinomas) | .523 | 1.401 | .498 | 3.941 |
| Morphology (Other malignancies) | .961 | .000 | .000 | 3.058E+205 |

Supplementary table 8: Population-based studies: An overview on available data

Incidence per 100,000

| **Country** | | **period** | **n** | **Basal cell carcinoma**  **(m/w)** | **Squamous cell carcinoma** | **Melanoma** | **other** | **Study** |
| --- | --- | --- | --- | --- | --- | --- | --- | --- |
| Germany | | 2009 – 2015 | 42,710 | 7.4 / 7.0 | 1.2 / 0.8 | 0.1 | N/A | current study |
| Ireland | | 2005 – 2015 | 4,824 | 15.9 / 13.5 | 2.1 / 1.4 | 0.1 | N/A | Quigley et al. 2019 |
| England | | 2000 – 2010 / 2000 – 2014 | 33,610 /  4,022 | 4.5 / 4.5 | 0.6 | N/A | N/A | Saleh et al. 2017, Wawrzynski et al. 2018 |
| South-western Finland | | 1995 – 1997 | 192 | 2.9 | N/A | N/A | N/A | Saari et al. 2001 |
| Finland | | 1953 – 1997 | 6,241 | 2.4 / 2.2 | N/A | N/A | N/A | Paavilainen et al. 2005 |
| Minnesota, USA | | 1976 – 1990 | 174 | 14.4 | 1.4 | 0.08 | N/A | Cook et al. 1999 |
| Hong Kong | | 2009 | 36 | 0.23 | N/A | N/A | N/A | Mak et al. 2011 |
| Florida, USA | | 1981 – 1994 | 206 | N/A | N/A | 0.06 (white males > 20) | N/A | Margo et al. 1998 |
| SEER 18, USA | | 1975-2016 | 1,397 | N/A | N/A | 0.039 | N/A | Shan et al. 2020 |
|  | **Study with non-melanoma eyelid cancers** | | | | | | | |
| Bulgaria | | 2000 – 2015 | 2.9 / 2.4 | | | | | Zlatarova et al. 2020 |
|  | **Studies with all eyelid cancers** | | | | | | | |
| Singapore | | 1968 – 1995 | 0.7 / 0.6 | | | | | Lee et al. 1999 |
| Singapore | | 1996 – 2008 | 0.5 | | | | | Lim et al. 2012 |
| Taiwan | | 1979 – 1999 | 0.3 | | | | | Lin et al. 2006 |
| Korea | | 2016 | 0.6 | | | | | Jung et al. 2020 |

Supplementary Figure 1: Crude Incidence Ratio CIR, and Age-Adjusted Incidence Ratio Per Year. Blue (men), Red (Women), Orange (Both)


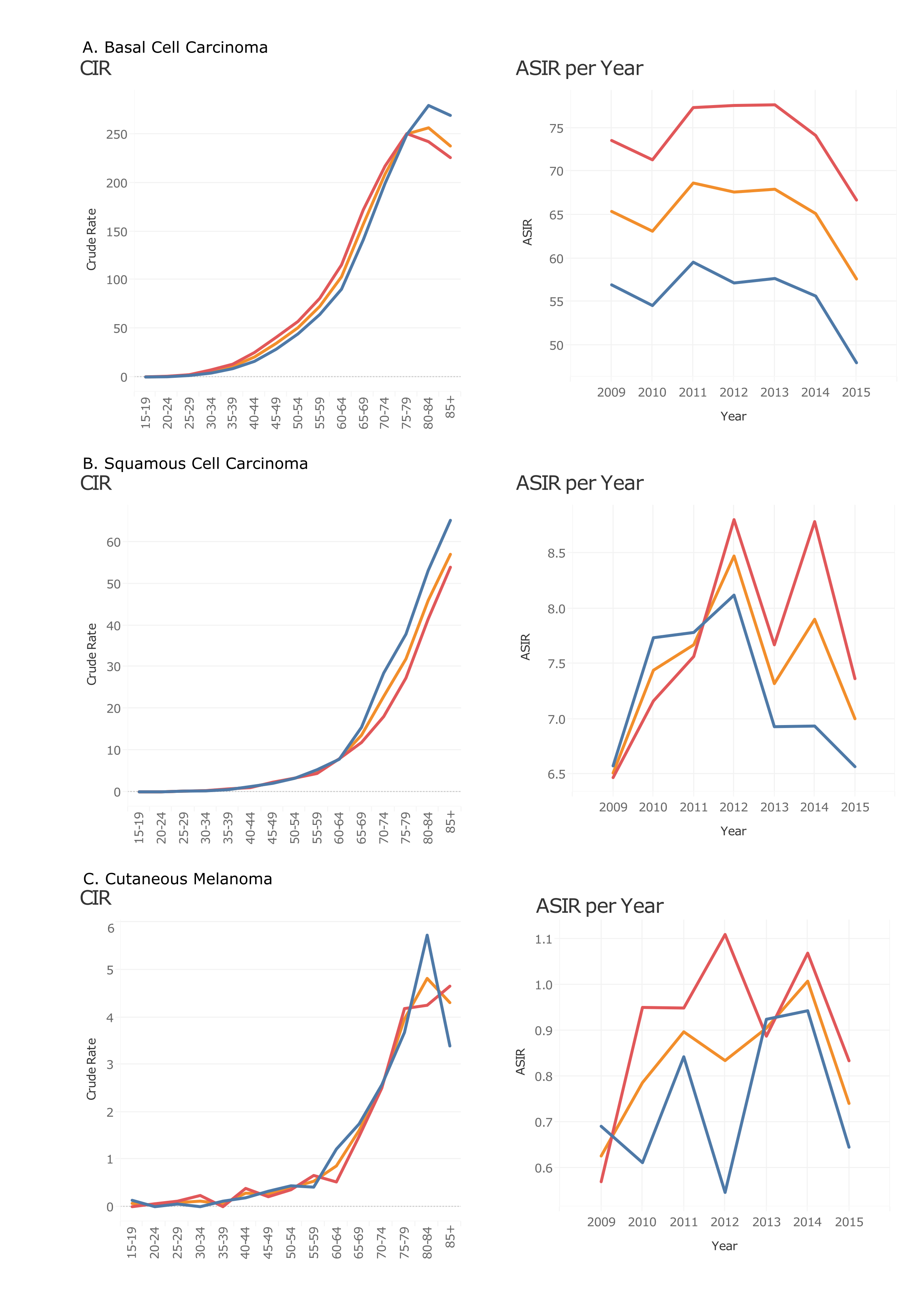


Supplementary Figure 2: Testing Cox-Regression Model for Overall survival


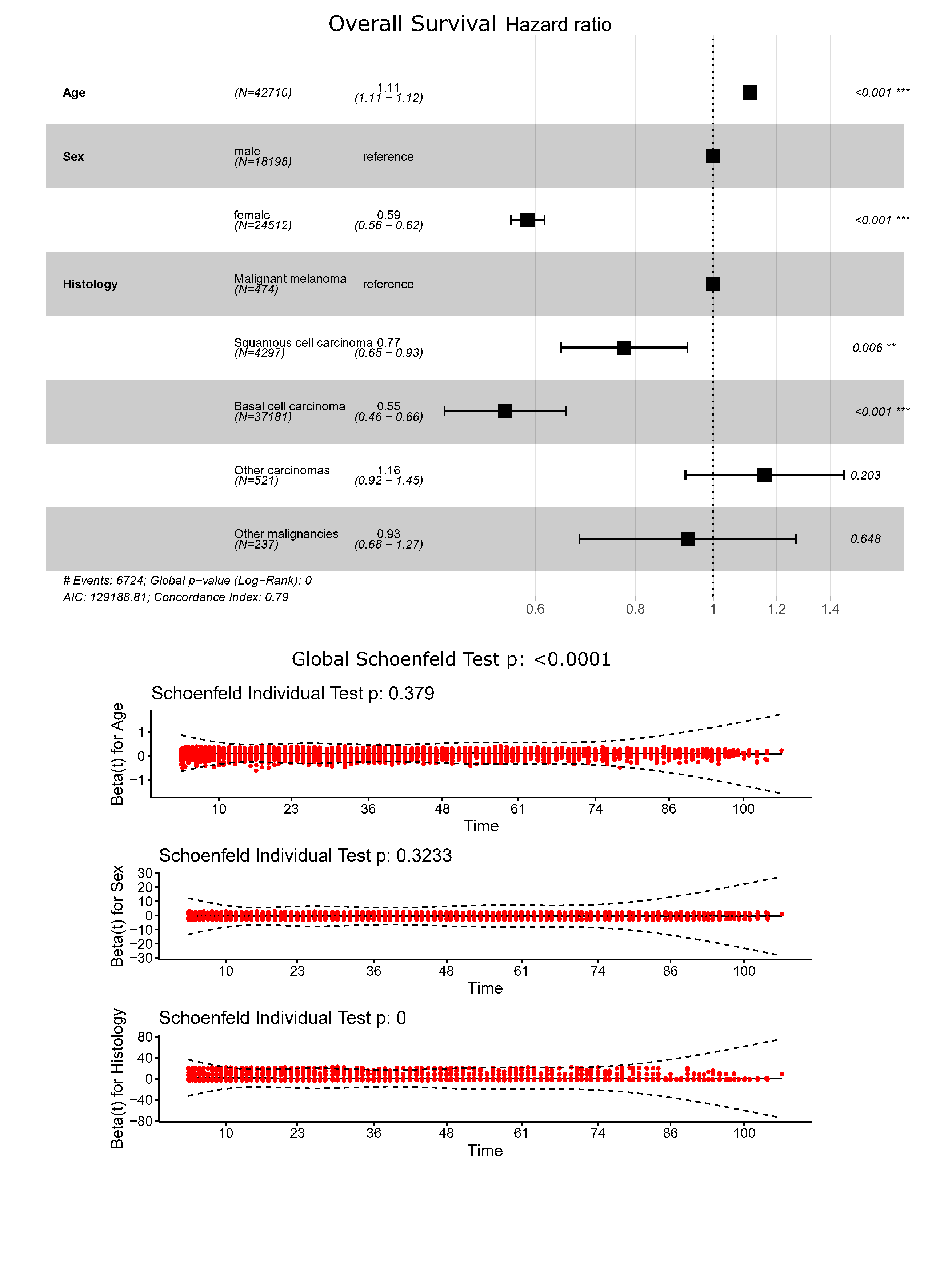

Supplement: sj-docx-1-ejo-10.1177_11206721221125018 - Supplemental material for The epidemiology of adults' eyelid malignancies in Germany between 2009 and 2015; An analysis of 42,710 patients' data [file sj-docx-1-ejo-10.1177_11206721221125018.docx]
